# Amino acid δ^15^N differences consistent with killer whale ecotypes in the Arctic and Northwest Atlantic

**DOI:** 10.1371/journal.pone.0249641

**Published:** 2021-04-02

**Authors:** Cory J. D. Matthews, Jack W. Lawson, Steven H. Ferguson

**Affiliations:** 1 Fisheries and Oceans Canada, Winnipeg, Manitoba, Canada; 2 Fisheries and Oceans Canada, St. John’s, Newfoundland, Canada; Senckenberg Gesellschaft fur Naturforschung, GERMANY

## Abstract

Ecotypes are groups within a species with different ecological adaptations than their conspecifics. Eastern North Pacific (ENP) killer whale (*Orcinus orca*) ecotypes differ in their diet, behavior, and morphology, but the same is not known for this species in the eastern Canadian Arctic (ECA) and Northwest Atlantic (NWA). Using compound-specific stable isotope analysis (CSIA) of amino acids (AAs), we compared δ^15^N patterns of the primary trophic and source AA pair, glutamic acid/glutamine (Glx) and phenylalanine (Phe), in dentine collagen of (1) sympatric ENP killer whale ecotypes with well-characterized diet differences and (2) ECA/NWA killer whales with unknown diets. δ^15^N_Glx-Phe_ was significantly higher in the ENP fish-eating (FE) than mammal-eating (ME) ecotype (19.2 ± 0.4‰ vs. 13.5 ± 0.7‰, respectively). Similar bimodal variation in δ^15^N_Glx-Phe_ indicated analogous dietary divisions among ECA/NWA killer whales, with two killer whales having higher δ^15^N_Glx-Phe_ (16.5 ± 0.0‰) than the others (13.5 ± 0.6‰). Inferences of dietary divisions between these killer whales were supported by parallel differences in threonine δ^15^N (–33.5 ± 1.6‰ and –40.4 ± 1.1‰, respectively), given the negative correlation between δ^15^N_Thr_ and TP across a range of marine consumers. CSIA-AA results for ECA/NWA whales, coupled with differences in tooth wear (a correlate for diet), are consistent with ecotype characteristics reported in ENP and other killer whale populations, thus adding to documented ecological divergence in this species worldwide.

## Introduction

Ecotypes are individuals or groups within a species with unique ecological adaptations, with accompanying differences in behavior, morphology, or physiology [1,2]. Killer whale (*Orcinus orca*) ecotypes were first identified in the eastern North Pacific (ENP) [3], where a fish-eating (FE) ecotype known as ‘residents’, which forage almost exclusively on salmon, is sympatric with a mammal-eating (ME) ecotype known as ‘transients’ or Bigg’s killer whales [4–6]. A second fish-eating ecotype termed ‘offshores’, whose diet includes sharks and bony fishes, has also been identified [7,8]. Killer whale ecotypes exert different top-down impacts on community structure (e.g., [9]) and are subject to different bottom-up constraints on population demographics [10,11], while their unique foraging adaptations have driven cultural and reproductive isolation that has been defined as incipient speciation [12,13].

Killer whale populations with dietary and morphological differences consistent with ecotypes have now been identified in the Southern Ocean [14–16], the Northwest Pacific [17–19], and the Northeast Atlantic, where generalist (Type 1) and specialist (Type 2) types occur [20–22]. Comparatively little is known about ecological divergence among killer whales in the northwest Atlantic (NWA), where killer whales off the coast of Newfoundland and Labrador have been observed pursuing or feeding on marine mammals and fish, including odontocetes, mysticetes, and seals, as well as herring and tuna [23,24]. Further north, killer whales have been observed hunting only marine mammals during their seasonal occupancy of the Eastern Canadian Arctic (ECA) [25,26], although some killer whales forage on fish off neighboring western Greenland [27].

Despite their broad reported prey base, Matthews and Ferguson [28] inferred individual dietary specialization among ECA/NWA killer whales using compound specific stable isotope analysis (CSIA) of amino acids (AA). Application of CSIA-AA in trophic studies is predicated upon the differential ^15^N fractionation of trophic AAs, which undergo trophic ^15^N enrichment, and source AAs, which are assumed to retain basal food web δ^15^N values. Empirical studies measuring ^15^N enrichment of multiple AAs with trophic transfer in food webs comprising algae, zooplankton, and fish [29–31] showed glutamic acid (Glx; see Methods) exhibited the most consistent and highest ^15^N enrichment with each trophic transfer (~8‰), while phenylalanine (Phe) underwent only a slight increase of ~0.4‰ with each trophic transfer. The relative difference in δ^15^N between these trophic and source AAs (δ^15^N_Glx-Phe_) thus allows for calibration of consumer trophic position (TP; [30,31]). Matthews and Ferguson [28] therefore interpreted two of the 13 sampled ECA/NWA whales with higher δ^15^N_Glx-Phe_ as having foraged at a higher TP, in line with conventions established from earlier CSIA-AA studies that demonstrated a positive correlation between δ^15^N_Glx-Phe_ and TP [29–31]. They further speculated these two killer whales may have fed primarily on sharks, given they had pronounced apical tooth wear that has been associated with shark diets in other killer whale populations [8].

Controlled diet studies [32–35] and meta-analyses [36,37] of a broad range of marine fish, birds, and mammals, including cetaceans [38], have since highlighted considerable variation in δ^15^N_Glx-Phe_ that is unrelated to TP. Variation in δ^15^N_Glx-Phe_ has been attributed to mechanisms affecting trophic ^15^N enrichment of glutamic acid, including the mode of nitrogen excretion and protein quantity and composition [32,33,35,36]. A small number of studies have also reported variation in trophic ^15^N enrichment of Phe, potentially reflecting its catabolism as an energy source vs. direct routing to growth [39]. Recent studies have also shown that δ^15^N of threonine, which is unique among AAs in its progressive ^15^N *depletion* with TP [32,36,40–42], is more strongly correlated with TP than any other AA [36]. δ^15^N_Thr_ alone may be appropriate for TP reconstructions [43].

Here we re-visit assumptions made by Matthews and Ferguson [28] with expanded sampling and re-analysis, including addition of genetically assigned fish-eating (FE) and mammal-eating (ME) killer whale ecotypes from the eastern North Pacific (ENP), along with threonine δ^15^N data from the ECA/NWA killer whales. We hypothesized that broad dietary differences between killer whale ecotypes would lead to measurable δ^15^N_Glx-Phe_ differences that could serve as a diagnostic framework with which the ECA/NWA population(s) could be re-assessed. Similar bimodal variation in δ^15^N_Glx-Phe_ observed between the known ENP ecotypes was also observed among ECA/NWA killer whales, indicating a similar degree of ecological divergence within this understudied population(s). Parallel differences in δ^15^N_Thr_ among the ECA/NWA whales support this interpretation, underscoring the utility of CSIA-AA as a new approach for characterizing killer whale ecotypes.

## Methods

### Sample collection

**Fig 1:**
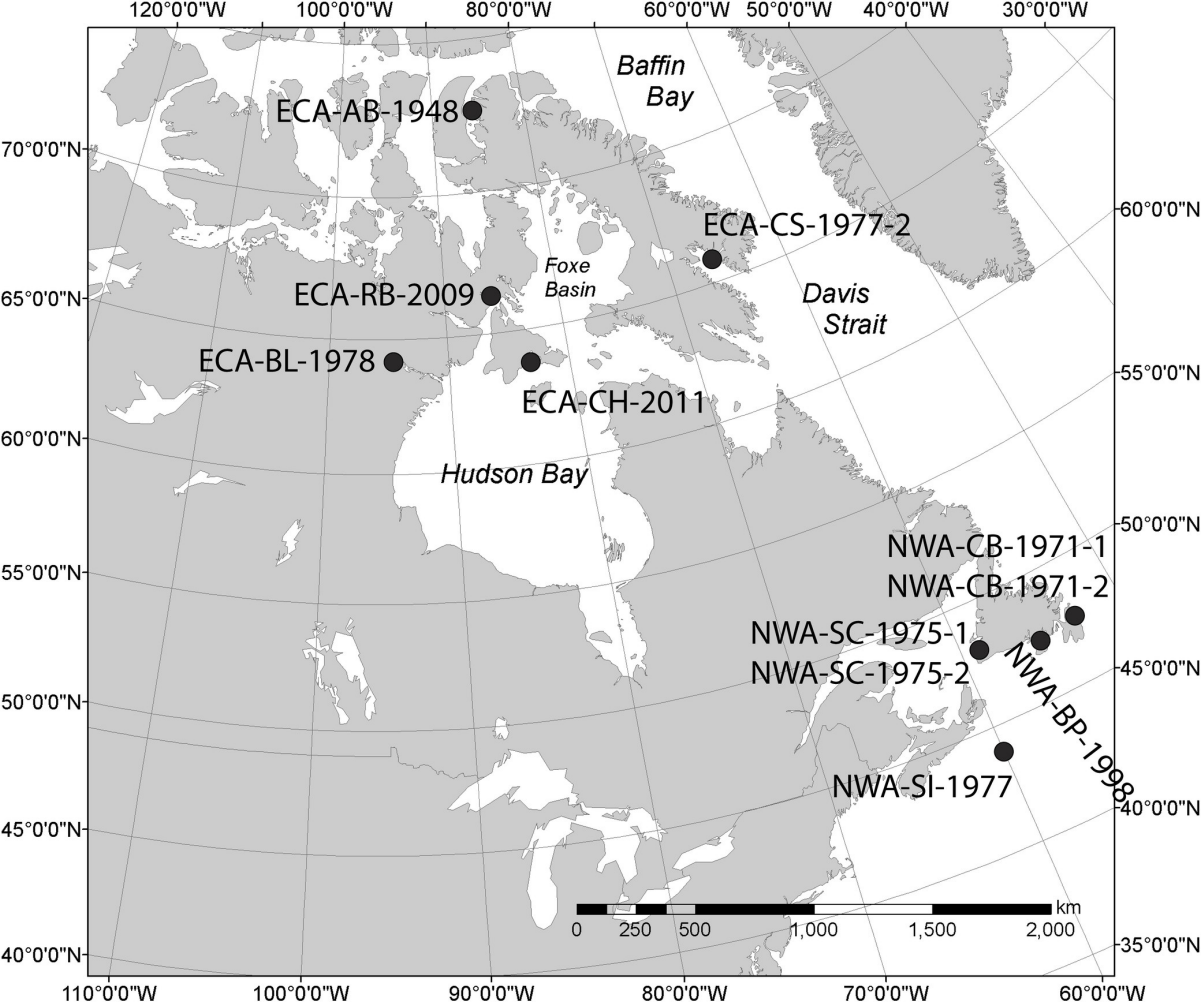
Locations of stranded killer whales in the eastern Canadian Arctic (ECA) and Northwest Atlantic (NWA) included in this study (specimen ID numbers match those presented in Table 1).

Teeth from genetically assigned FE (n = 3) and ME killer whale ecotypes (n = 4) that stranded around Vancouver Island and the lower British Columbia mainland (n = 7), and from killer whales that stranded at various locations in the ECA (n = 5) and NWA (n = 6) (Fig 1), were acquired for destructive sampling from museum and government collections (Table 1). The distinct fish and mammal diets of the ENP ecotypes have been characterized through decades of field observations [4–6] and various chemical diet proxies, including stable isotopes, fatty acids, and contaminants [44–46]. Sex and morphometric data (e.g., body length) were available for a limited number of specimens (Table 1). Animal use protocol approval was not required, as all specimens were from archived natural history collections.

**Table 1:** Stranding location and other data (estimated age, sex, and body length) of killer whale (*Orcinus orca*) ecotypes from the eastern North Pacific (ENP) and killer whales from the eastern Canadian Arctic (ECA) and Northwest Atlantic (NWA) included in this study.

	Specimen ID	Institution collection	Stranding location and year	Sex	Age (yr)	Length (cm)
**Fish-eating (FE) ENP Ecotype (residents)**	16814	Royal British Columbia Museum	Vancouver Island, BC, 1989	M	15–16	610
	16006	Royal British Columbia Museum	Vancouver Island, BC, 1986	F	adult	630
	8386	Royal British Columbia Museum	Vancouver Island, BC, 1973	M	adult	488
**Mammal-eating (ME) ENP Ecotype (transients)**	10001	Royal British Columbia Museum	Lower mainland, BC, 1979	M	adult	699
	10674	Royal British Columbia Museum	Vancouver Island, BC, 2013	U	adult	550
	10402	Royal British Columbia Museum	Vancouver Island, BC, 1981	M	immature	450
	F76-31 3	Royal British Columbia Museum	Vancouver Island, BC, 1976	M	adult	681
**ECA**	ECA-AB-1948	Manitoba Museum	Arctic Bay, NU, 1948	U	31*	not measured
	ECA-CS-1977-2	Fisheries and Oceans Canada	Cumberland Sound, NU, 1977	U	18*	not measured
	ECA-BL-1978	Fisheries and Oceans Canada	Baker Lake, NU, 1978	M	17*	not measured
	ECA-CH-2011	Fisheries and Oceans Canada	Coral Harbour, NU, 2011	M	35*	not measured
	ECA-RB-2009*	Fisheries and Oceans Canada	Naujaat (Repluse Bay), NU, 2009	F	28*	570
**NWA**	NWA-SC-1975-2	Canadian Museum of Nature	Ship Cove, NL, 1975	M	23*	610
	NWA-SC-1975-1	Canadian Museum of Nature	Ship Cove, NL, 1975	M	20*	742
	NWA-CB-1971-1	Canadian Museum of Nature	Conception Bay, NL, 1971	M	31*	755
	NWA-CB-1971-2	Canadian Museum of Nature	Conception Bay, NL, 1971	F	29*	618
	NWA-SI-1977	Nova Scotia Museum	Sable Island, NS, 1977	U	13*	not measured
	NWA-BP-1998*	Fisheries and Oceans Canada	Burin Peninsula, NL, 1998	F	5*	not measured

*estimated from counts of annual growth layer groups (GLGs) [28].

ENP ecotypes were genetically assigned (G. Hanke, Royal British Columbia Museum, Personal Communication), and ECA/NWA data were originally presented in Matthews and Ferguson [28].

‘Whole-tooth’ dentine samples were micromilled from longitudinal midline sections along paths that traversed all annual growth layers beyond the third GLG, as previous studies of both ENP and ECA/NWA killer whale teeth showed little within-tooth SI variation after weaning by age 3 [28,46]. Collagen was isolated from dentine powder using several 12-hr washes in 0.25 N HCl at 4°C followed by repeated rinses with distilled water, and then freeze-dried with no additional processing prior to analysis [28,46]. Atomic C:N (mean ± SD = 3.26 ± 0.01; range = 3.24 to 3.28) was within the range of unaltered collagen [47].

### Compound specific stable isotope analysis of amino acids (CSIA-AA)

The ECA/NWA samples analysed previously using a different protocol [28] were re-analysed along with the ENP samples using the same protocol for consistency. Approximately 3 mg of each dentine collagen sample was acid hydrolysed in 6M HCl for 70 min at 150°C under a N_2_ headspace, and derivatized using methoxycarbonylation esterification following Walsh et al. [48] and Yarnes and Herszage [49]. While pH-dependent fractionation during methoxycarbonylation esterification can produce two Glu derivatives with different δ^15^N values, all analyses were conducted under pH conditions (<< 1) that produce a single derivative (pyroglutamic acid) that retains the δ^15^N of the original parent Glu [48,49]. We use the IUPAC-accepted terminology Glx (Glu + glutamine) for the AA mixture that results from the conversion of glutamine to Glu during derivatization.

δ^15^N of derivatized AAs was measured using a Trace Ultra gas chromatograph with a DB-23 column (30 m length, 0.25 mm outer diameter, 0.25 mm film; Agilent Technologies) coupled to a Thermo Delta V Plus via a GC IsoLink. Following Yarnes and Herszage [49], two AA mixtures previously calibrated to atmospheric N_2_ were used in calibration and scale-normalization procedures, while a third AA mixture served as the primary quality control reference material. Standard deviations of replicate measurements of co-measured reference compounds not used in calibrations (baleen, n = 12; fish muscle, n = 12) were 0.81‰ for δ^15^N_Phe_ and 0.76‰ for δ^15^N_Glx_, and those based on duplicate measures of each sample ranged from 0.06 to 1.38‰ for δ^15^N_Phe_ and from 0.02 to 1.10‰ for δ^15^N_Glx_.

Threonine δ^15^N values are reported for only the ECA/NWA killer whale samples, previously unpublished but available from their prior analysis using acetylation-esterification derivatization [28]. The methoxycarbonylation esterification method used in this study results in the co-elution of threonine with aspartic acid without full resolution, which precluded threonine δ^15^N measurement in the known ENP ecotypes. δ^15^N_Thr_ of the ECA/NWA whales is reported with a precision <0.88 ‰.

### Bulk collagen stable isotope analysis

Bulk dentine collagen δ^15^N and δ^13^C of the ENP ecotypes were measured to provide additional information to assist interpretations of CSIA-AA results, primarily via comparison with published values of potential marine mammal prey in the ENP (see Discussion). Samples were analysed using continuous flow isotope ratio mass spectrometry (CF-IRMS) at the University of California-Davis Stable Isotope Facility (bulk SI values of the ECA/NWA killer whale samples were analysed previously using similar standard protocols; see Matthews and Ferguson [28]). δ^15^N and δ^13^C, defined as (R_sample_—R_standard_)/R_standard_)*1,000, where R is the ratio of the abundance of the heavy to light isotope, were measured in approximately 1 mg samples and normalized to atmospheric nitrogen (Air) and Vienna Pee Dee Belemnite carbonate, respectively, using four laboratory reference materials of known isotopic composition (δ^15^N range: –6.80 to 41.13‰; δ^13^C range: –27.76 to –16.65‰). Standard deviations of repeated measures of reference materials (bovine liver, n = 2; enriched alanine, n = 8; glutamic acid, n = 3; and nylon 6, n = 21) ranged from 0.02 to 0.12‰ for δ^15^N and 0.02 to 0.08‰ for δ^13^C.

### Data analysis

Data normality could not be assessed due to small sample sizes, so the nonparametric Kruskal-Wallis rank sum test was used to assess δ^15^N_Glx-Phe_ differences between ecotypes and among ECA/NWA killer whales with purported dietary differences. All analyses were conducted using R software [50].

## Results

### Known ENP ecotypes

**Fig 2:**
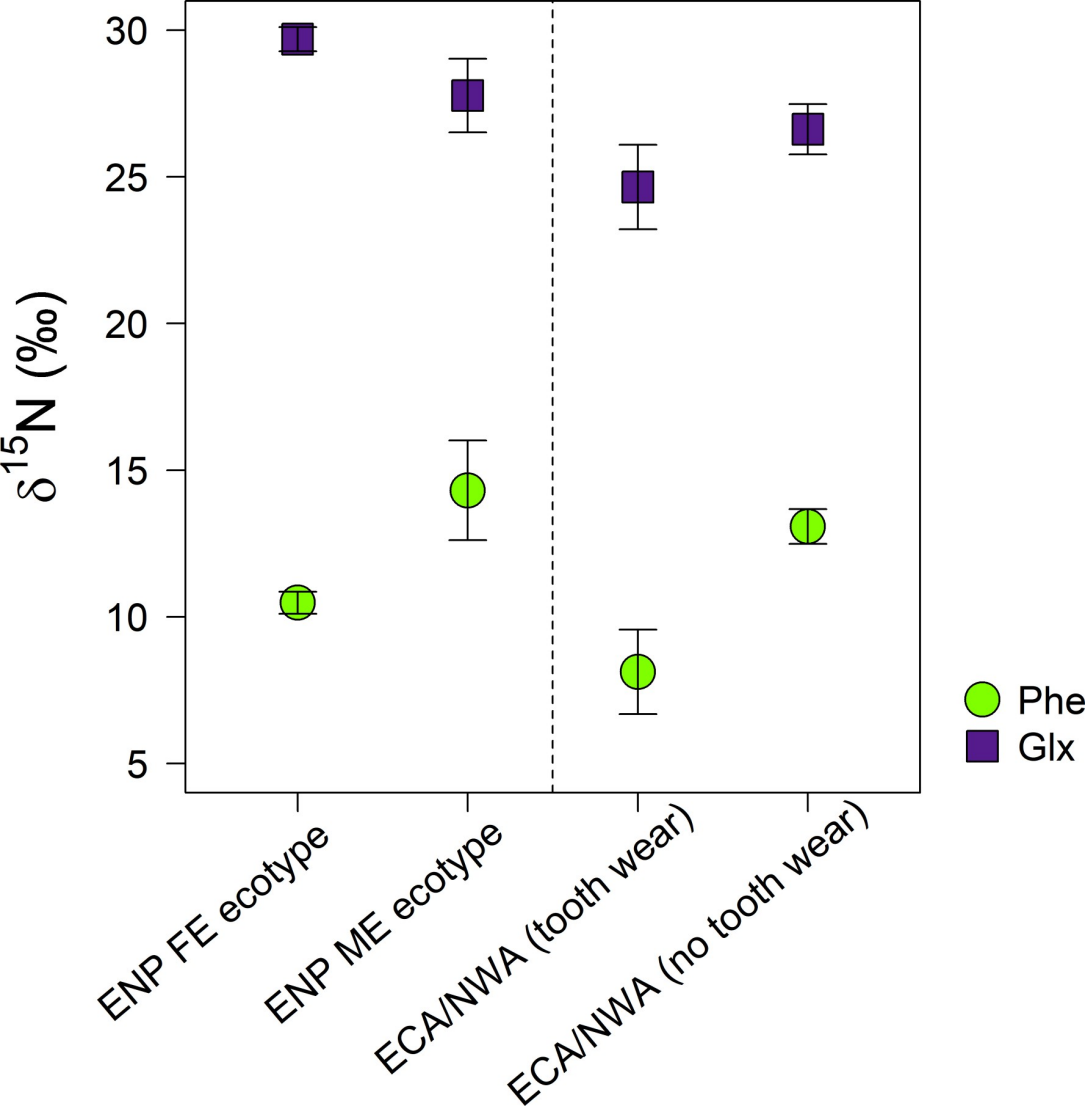
δ^15^N_Phe_ (circles) and δ^15^N_Glx_ (squares) of eastern North Pacific (ENP) killer whale ecotypes (fish-eating residents and mammal-eating transients) and eastern Canadian Arctic/Northwest Atlantic (ECA/NWA) killer whales with tooth wear and no tooth wear (error bars = standard deviation). Similar relative differences in δ^15^N_Glx-Phe_ among the ECA/NWA groups as the ENP ecotypes suggest similar dietary divisions.

δ^15^N_Glx-Phe_ values were higher in FE (19.2 ± 0.4‰) than ME (13.5 ± 0.7‰) killer whales (Fig 2, Table 2; Kruskal-Wallis rank sum test, chi-squared = 4.5, df = 1, *p* < 0.05).

**Table 2:** Bulk δ^15^N and δ^13^C values and phenylalanine (Phe), glutamic acid/glutamine (Glx), and threonine (Thr) δ^15^N values (‰) measured in dentine collagen of killer whale (*Orcinus orca*) ecotypes from the eastern North Pacific (ENP), and killer whales from the eastern Canadian Arctic (ECA) and Northwest Atlantic (NWA) with unknown diets.

	Whale ID	Bulk δ^15^N	Bulk δ^13^C	δ^15^N_Phe_	δ^15^N_Glx_	δ^15^N_Glx-Phe_	δ^15^N_Thr_
**Fish-eating (FE) ENP Ecotype (residents)**	16814	17.85	–12.17	10.66 ± 1.0	30.17 ± 0.3	19.50	not measured
	16006	17.63	–11.43	10.74 ± 0.2	29.45 ± 0.5	18.71	not measured
	8386	17.57	–12.59	10.05 ± 0.1	29.46 ± 0.9	19.40	not measured
**Mean ± SD**				**10.48 ± 0.38**	**29.69 ± 0.41**	**19.21 ± 0.43**	not measured
**Mammal-eating (ME) ENP Ecotype (transients)**	10001	19.77	–11.41	15.20 ± 0.1	28.56 ± 0.8	13.36	not measured
	10674	15.59	–15.17	11.96 ± 0.2	25.90 ± 0.9	13.95	not measured
	10402	20.46	–13.08	15.84 ± 0.4	28.35 ± 1.0	12.51	not measured
	F76-31 3	19.91	–11.26	14.24 ± 0.6	28.27 ± 0.5	14.03	not measured
**Mean ± SD**				**14.31 ± 1.70**	**27.77 ± 1.25**	**13.46 ± 0.70**	not measured
**ECA**	ECA-AB-1948	17.8 ± 0.6	–13.4 ± 0.3	13.87 ± 1.4	28.63 ± 0.0	14.76	–41.12 ± 0.6
	ECA-CS-1977-2	18.3 ± 0.3	–14.4 ± 0.4	13.79 ± 0.1	26.64 ± 0.2	12.85	–39.12 ± 0.9
	ECA-BL-1978	17.9 ± 0.6	–14.1 ± 1.4	12.77 ± 0.5	26.02 ± 1.0	13.25	–39.16 ± 0.4
	ECA-CH-2011	18.1 ± 0.6	–14.4 ± 0.2	13.35 ± 0.1	26.93 ± 1.0	13.58	–41.19 ± 0.3
	ECA-RB-2009*	**15.1 ± 0.8**	–**15.1 ± 0.4**	**9.14** ± 1.3	**25.66** ± 0.5	**16.52**	**–35.12** ± 0.3
**NWA**	NWA-SC-1975-2	17.2 ± 0.6	–13.9 ± 0.4	13.04 ± 1.3	26.03 ± 0.3	12.99	–40.99 ± 0.4
	NWA-SC-1975-1	17.3 ± 0.7	–13.8 ± 0.4	12.90 ± 0.7	26.80 ± 0.8	13.91	–40.91 ± 0.7
	NWA-CB-1971-1	16.5 ± 0.5	–13.7 ± 0.2	12.24 ± 1.0	25.62 ± 0.9	13.39	–38.46 ± 0.4
	NWA-CB-1971-2	16.6 ± 0.5	–14.0 ± 0.3	12.28 ± 0.2	26.32 ± 1.1	14.04	–40.28 ± 0.7
	NWA-SI-1977	17.1 ± 0.3	–14.4 ± 0.3	13.45 ± 0.4	26.60 ± 0.6	13.15	–42.09 ± 0.6
	NWA-BP-1998*	**13.7 ± 0.5**	–**15.3 ± 0.3**	**7.10** ± 0.5	**23.63** ± 0.3	**16.53**	**–31.89** ± 0.4

*suspected shark-eater based on tooth wear [28].

Bulk δ^15^N and δ^13^C values of the ECA and NWA whales are averages (± SD) of individual dentine growth layer groups [28]. All δ^15^N_Glx_ and δ^15^N_Phe_ values are averages (± SD) of duplicate measurements, while δ^15^N_Thr_ are averages (± SD) of triplicate measurements. δ^15^N_Glx-Phe_ is considered a relative trophic index.

Bulk dentine collagen δ^15^N values of the three FE killer whales ranged from 17.6 to 17.9‰. Those of the four ME killer whales ranged from 15.6 to 20.5‰, or from 19.8 to 20.5‰ excluding the lowest value (Table 2). Bulk dentine collagen δ^13^C values of the three FE killer whales ranged from –12.6 to –11.4‰, and those of the three ME with similar δ^15^N values ranged from –13.1 to –11.3‰. The ME killer whale with the lowest bulk δ^15^N also had the lowest δ^13^C value (–15.7‰; Table 2).

### ECA/NWA killer whales

Bimodal variation in δ^15^N_Glx-Phe_ was also observed among the ECA/NWA killer whales (Fig 2). δ^15^N_Glx-Phe_ values of two ECA/NWA killer whales (both 16.5‰) were higher than those of the other ECA/NWA killer whales (13.5 ± 0.6‰) (Table 2) (Kruskal-Wallis rank sum test, chi-squared = 4.5, df = 1, *p* < 0.05). The two killer whales with the higher δ^15^N_Glx-Phe_ had higher δ^15^N_Thr_ values (–33.5 ± 1.6‰) than those with lower δ^15^N_Glx-Phe_ (–40.4 ± 1.1‰; Table 2) (Kruskal-Wallis rank sum test, chi-squared = 4.5, df = 1, *p* < 0.05).

## Discussion

Ecological divergence of the two ENP killer whale ecotypes was discernable using CSIA-AA, albeit with results that challenge conventions established from previous CSIA-AA studies of marine consumers (discussed in detail in Matthews et al. [38]). Conventional interpretation of the lower δ^15^N_Glx-Phe_ values of the ME ecotype would have them feeding at a lower TP than the FE ecotype, which we consider implausible. Decades of observational studies indicate baleen whales, the only marine mammals that generally occupy a lower TP than the salmon (*Oncorhynchus* spp.) prey of FE killer whales [51,52], are not the primary prey of ME killer whales in the ENP [4,5,45]. Although consumption of gray whale (*Eschrichtius robustus*) calves and yearlings and minke whales (*Balaenoptera acutorostrata*) is seasonally important off Alaska [45,53], the vast majority of successful kills (89 to 100%) over 20 years of study off British Columbia, Washington, and Alaska involved pinnipeds and porpoises [4,5,54]. Moreover, killer whales are known to consume relatively small proportions (e.g., the tongue and ventral skin) of baleen whale kills [55,56].

Bulk SI values corroborate long-term observational studies that ME killer whales in the ENP feed primarily on pinnipeds and porpoises, not baleen whales. Continual dentine deposition [57] in whole-tooth samples analysed here integrates long-term diet that would attenuate isotopic signals related to seasonal consumption of prey. After adjustment for collagen-specific trophic enrichment (~3‰; [58]) to allow for direct, tissue-specific comparison with potential prey, the three ME killer whales with the highest adjusted values (16.8 to 17.5‰) are considerably higher than bulk bone collagen δ^15^N values of both gray and minke whales in the ENP (14.2 ± 0.7 and 14.4 ± 0.8‰, respectively; [46,58], but fall within the range of bone collagen and dentine δ^15^N of ENP pinniped (15.7 to 18.6‰; [59–61] and harbor porpoise (15.7 ± 0.7‰; [62]).

The adjusted bulk δ^15^N value (~12.6‰) of the fourth (outlier) ME killer whale falls well below the δ^15^N values of potential marine mammal prey in the ENP, including baleen whales. This, along with its lower bulk δ^13^C values, suggests it fed primarily within a different, isotopically distinct, region of the ENP. This interpretation is supported by the fact that both its δ^15^N_Phe_ and δ^15^N_Glx_ values were offset by the same relative amounts as the other three ME killer whales, indicating it foraged at a similar TP, but within a region characterized by lower baseline isotope values. Temporal isotopic variation over the decades separating specimen collection (see Table 1) could likewise lead to different bulk but similar relative AA-specific SI differences among individuals, although regional baseline δ^15^N has been relatively stable over that timeframe [61]. Genetics analysis confirmed this whale possessed the AT1 haplotype (Lance Barrett-Lennard, Vancouver Aquarium, Personal Communication), which is present in transient-type killer whales as far west as Russia [63]. Marine (bulk) δ^15^N and δ^13^C values decrease along an east to west gradient in the Bering Sea [64], and pinniped bone collagen δ^15^N and δ^13^C are lower (by ~2–4‰ and 2–3‰, respectively) off the outer Aleutians than the Gulf of Alaska and coasts of British Columbia and California [61,65]. Long distance movements spanning thousands of kilometers have been documented in ENP transients [66]; a more northwestern distribution could therefore possibly account for this killer whale’s lower bulk isotope values.

Our comparison of genetically-identified fish and mammal-eating ENP killer whale ecotypes thus yielded results that are inconsistent with previous CSIA-AA studies, the details of which are discussed in Matthews et al. [38]. Nevertheless, what is relevant to the objectives of the present study is that the AA-specific δ^15^N differences between the ecotypes were consistent, thereby providing a diagnostic framework with which to interpret similar bimodal differences in δ^15^N_Glx-Phe_ among ECA/NWA killer whales. Like the FE ecotype, two ECA/NWA killer whales had significantly higher δ^15^N_Glx-Phe_ values than the other ECA/NWA killer whales. Matthews and Ferguson [28] hypothesized these two whales may have fed primarily on sharks owing to their pronounced tooth wear similar to that of the offshore killer whale ecotype in the ENP [8], and the typically high TPs of sharks [67] is consistent with studies showing a positive correlation between δ^15^N_Glx-Phe_ and TP (e.g., [29–31]).

In light of the results from the present study, however, we conclude that these killer whales most likely fed at a *lower* TP than the others. Severe tooth wear has also been attributed to suction feeding on forage fish such as herring (*Clupea harengus*) or mackerel (*Scomber scombrus*) by killer whales in the northeast Atlantic [20]. Killer whales have been observed in association with other predators feeding on herring off Newfoundland [24]. Given recent evidence that δ^15^N_Glx-Phe_ broadly reflects feeding guild [36,38], it is also possible that the intermediate δ^15^N_Glx-Phe_ values of the two ECA/NWA whales (both 16.5‰) relative to the FE and ME ENP ecotypes (19.2 ± 0.4‰ and 13.5 ± 0.7‰, respectively) reflects a mixed diet comprising both fish and mammals. Primarily herring-eating killer whales from other populations in the North Atlantic consume variable proportions of grey seals *Halichoerus grypus* [20,22], as well as seasonally available Arctic marine mammals off southeast Greenland [68]. Unfortunately, samples from the offshore ENP ecotype, which would have allowed for comparison of δ^15^N_Glx-Phe_ resulting from a purported shark diet, were unavailable for this study.

The similar δ^15^N_Glx-Phe_ values of the remaining ECA/NWA killer whales (13.5 ± 0.6‰) and the ME ecotype (13.5 ± 0.7‰) suggests they also foraged primarily on marine mammals. This interpretation is consistent with observations in the ECA, where the majority of killer whale attacks are focused on narwhals (*Monodon monoceros*) and belugas (*Delphinapterus leucas*; [26]) and phocid seals [69]. Killer whales prey on a range of marine mammal species off the coast of Newfoundland [23,24], although, unlike in the ENP, minke whales appear to be their predominant prey [24]. Minke whales in the North Atlantic commonly feed on forage fish such as herring, capelin (*Mallotus villosus*), or mackerel [70,71], so killer whales feeding mainly on minke whales would occupy a higher TP than herring specialists. However, we note that remains of both minke whales and seals have been recovered from stomachs of killer whales off Newfoundland [23], and that seasonally-biased observations of predation [72] may not describe the full dietary breadth of killer whales. This is certain for killer whales observed in the Arctic during the relatively short open-water season, as the distributions of their Arctic marine mammal prey do not extend to the more southern limits of their range (see [73,74].

Parallel δ^15^N_Glx-Phe_ results between the ENP ecotypes and ECA/NWA killer whales suggest analogous dietary divisions, an interpretation that is supported, at least in general terms, by threonine δ^15^N differences among the ECA/NWA whales. Although the underlying biochemical mechanism for threonine’s progressive ^15^N depletion with TP is not well-understood [43], a review of over 47 marine teleost species spanning 2.5 TPs found δ^15^N_Thr_ displayed the most significant linear (*negative*) correlation with TP of any AA examined [36]. Given consistent ^15^N depletion of threonine on the order of 6‰ or higher with each trophic transfer [41,42], the approximately 7‰ higher mean δ^15^N_Thr_ of the two killer whales with higher δ^15^N_Glx-Phe_ is consistent with their having fed at a lower TP (by approximately one position, if the magnitude of ^15^N depletion is linear across all TPs/taxa). Unfortunately, corroboration with the known ENP ecotypes is not possible, as δ^15^N_Thr_ cannot be quantified using the methoxycarbonylation esterification derivatization method with which they were analysed (see Methods).

While dietary differences are a defining characteristic of killer whale ecotypes, ecotype designation requires additional assessment of movements, morphology, and genetics [2]. Although these requisite data are lacking for most whales in our sample, the Arctic killer whale with higher δ^15^N_Glx-Phe_ and δ^15^N_Thr_ and pronounced tooth wear (ECA-RB-2009) measured 570 cm (the other whale, NWA-BP-1998, was immature; Table 1). Populations of relatively small (< 6.5 m), piscivorous killer whales have been identified globally, including the ENP offshore ecotype [75], the Antarctic Type C killer whale [15], and the North Atlantic Type 1 killer whale [20,21]. Single morphologically similar specimens (~6 m long, with teeth worn to the gums) have also been reported off South Africa [76] and the Caribbean [77]. In contrast, three of the four killer whales with lower δ^15^N_Glx-Phe_ and δ^15^N_Thr_ with available length measurements (NWA-SC-1975-1, NWA-CB-1971-1, NWA-CB-1971-2) were larger than their respective sexes of piscivorous types described globally (the fourth, NWA-SC-1975-2, was an adult male aged 23 yr and measuring 610 cm; Table 1).

CSIA-AA has revealed similar bimodal variation between known ENP killer whale ecotypes and among killer whales from the ECA/NWA, adding to documented ecological divergence in this species worldwide. While limited morphological and genetics data warrant further study, amino acid-specific isotope results, coupled with morphological differences in tooth wear and available data on body size, are consistent with ecotype characteristics described in other populations. Our study of this relatively understudied population therefore contributes to ongoing discussions about ecological divergence in this species [2], while providing regionally relevant information for assessing the ecological influence of killer whales in both the ECA and NWA, where increasing numbers and range expansions have been documented over recent decades [78,79].
